# Integrating Triangle and Jaccard similarities for recommendation

**DOI:** 10.1371/journal.pone.0183570

**Published:** 2017-08-17

**Authors:** Shuang-Bo Sun, Zhi-Heng Zhang, Xin-Ling Dong, Heng-Ru Zhang, Tong-Jun Li, Lin Zhang, Fan Min

**Affiliations:** 1 School of Computer Science, Southwest Petroleum University, Chengdu 610500, China; 2 School of Science, Southwest Petroleum University, Chengdu 610500, China; 3 School of Mathematics, Physics and Information Science, Zhejiang Ocean University, Zhoushan 316022, China; Tianjin University, CHINA

## Abstract

This paper proposes a new measure for recommendation through integrating Triangle and Jaccard similarities. The Triangle similarity considers both the length and the angle of rating vectors between them, while the Jaccard similarity considers non co-rating users. We compare the new similarity measure with eight state-of-the-art ones on four popular datasets under the leave-one-out scenario. Results show that the new measure outperforms all the counterparts in terms of the mean absolute error and the root mean square error.

## Introduction

The distance measure is essential in machine learning tasks such as clustering [1, 2], classification [3, 4], image processing [5], and collaborative filtering [6–9]. Collaborative filtering (CF) through k-nearest neighbors (kNN) is a popular memory-based recommendation [10–12] schema. The key issue of CF scheme is how to calculate the similarity between users [6, 13] or items [14, 15]. Various types of similarity measures [16, 17] have been adopted or designed for this issue. State-of-the-art ones include Cosine [18], Pearson Correlation Coefficient (PCC) [6, 19], Jaccard [20], Proximity Impact Popularity (PIP) [21], New Heuristic Similarity Model (NHSM) [22] and so on. Naturally, new similarity measures providing better prediction ability are always desired.

**Fig 1:**
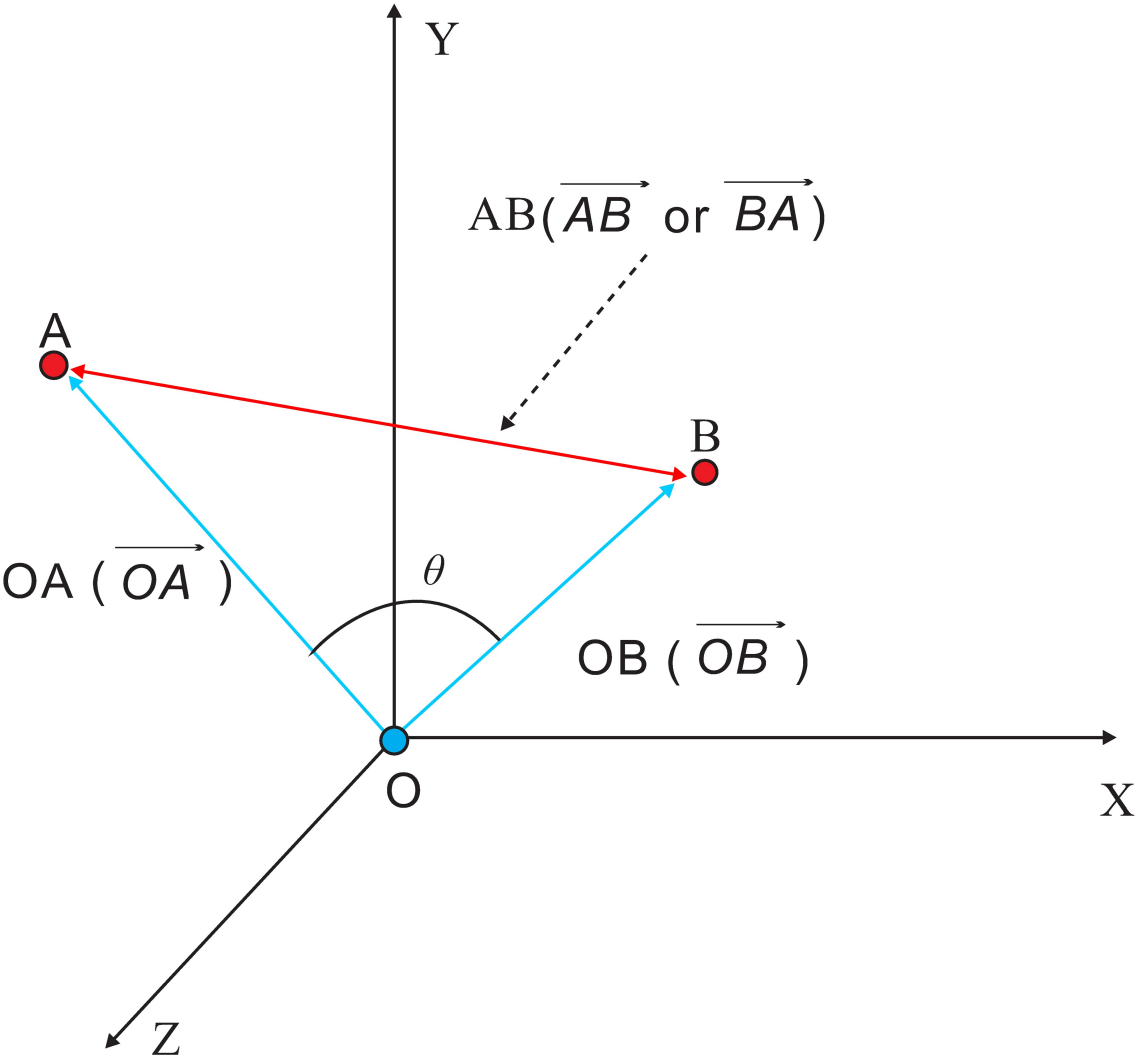
The Triangle in 3D space.

This paper proposes the Triangle multiplying Jaccard (TMJ) similarity. Only the item-based CF [14, 15, 23] will be considered since it performs better than the user-based [13, 24] one. As illustrated in Fig 1, the rating vectors of two items form a triangle in the space. The Triangle similarity is one minus the third divided by the sum of two edges corresponding to the vectors. Since it only considers the co-rating users, it is not good enough when used alone. Fortunately, the Jaccard similarity complements with it in that non co-rating users are considered. Therefore TMJ can take advantage of both Triangle and Jaccard similarities.

We compare TMJ with eight existing measures on four popular datasets under the leave-one-out scenario. These datasets include Movielens 100k, 1M, FilmTrust and EachMovie. The leave-one-out scenario is chosen because the result is not influenced by the division of the training/testing sets. Results show that the recommender system using TMJ outperforms all the counterparts in terms of the mean absolute error (MAE) and the root mean square error (RSME). Specifically, the MAE obtained on four datasets are 0.707, 0.671, 0.614 and 0.179, respectively.

In subsequent sections, we firstly review the basic concept of memory-based recommender system and eight popular similarity measures. Secondly we present the Triangle and TMJ similarities with a running example. Complexity analysis is also presented. Subsequently, we analyze the experimental results. Finally, we make our concluding remarks and indicate further work. All code files and data sets are available from the Github database (https://github.com/FanSmale/TMJSimilarity.git).

## Related work

In this section, we review eight similarity measures including the Cosine [18], PCC [6, 19], Constrained Pearson Correlation Coefficient (CPCC) [13], Jaccard [20], Bhattacharyya Coefficient (BC) [25, 26], Euclidean similarity (ES) [27, 28], PIP [21] and NHSM [22].

### Rating system

The user-item relationship is often expressed by a rating system. Let *U* = {*u*_1_, *u*_2_, …, *u*_*m*_} be the set of users of a recommender system and *I* = {*i*_1_, *i*_2_, …, *i*_*n*_} be the set of all possible items that can be recommended to users. Then the rating function is often defined as [29]

$$\begin{array}{c} r:U\times I\to R, \end{array}$$ (1)

where *R* is the rating domain used by the users to evaluate items.

For convenience, we let *r*_*u*,*i*_ be the rating of item *i* ∈ *I* evaluated by user *u* ∈ *U*, $r_{i}=\left(r_{u_{1},i},r_{u_{2},i}\cdots ,r_{u_{m},i}\right)$ be the rating vector of item *i*, and $\forall 1\leq j\neq q\leq n,C_{i_{j},i_{q}}$ be the set of co-rating users who have rated *i*_*j*_ and *i*_*q*_. Here we have the following example.

**Example 1** Table 1 *lists an example of rating system. R* = {1, 2, 3, 4, 5}, *where the numbers 1 through 5 represent the five rating levels; 0 indicates that the user has not rated the item. Given u*_4_ *and* *i*_2_, $r_{u_{4},i_{2}}=1$ *means that the rating of u*_4_ *to i*_2_ *is 1*. $r_{i_{1}}=\left\{4,5,4,2,4\right\}$ *is the rating vector of item i*_1_; $C_{i_{1},i_{3}}=\left\{u_{1},u_{3},u_{5}\right\}$ *is the set of co-rating users who have rated i*_1_ *and* *i*_3_.

**Table 1:** Rating system.

UID/IID	*i*_1_	*i*_2_	*i*_3_	*i*_4_	*i*_5_
*u*_1_	4	3	5	4	2
*u*_2_	5	3	0	0	4
*u*_3_	4	3	3	2	1
*u*_4_	2	1	0	1	2
*u*_5_	4	2	3	0	2

### The leave-one-out scenario

Leave-one-out cross validation is a general training/testing scenario for evaluating the performance of a recommender system as well as a classifier. Each time only one rating is used as the test set, and the remaining ratings are used as the training set. Different from split-in-two or 10-fold cross validation, the result is not influenced by the division of the training/testing sets.

An example of the leave-one-out scenario is listed as follows.

**Example 2** *Based on* Table 1, *we first leave* $r_{u_{1},i_{1}}$ *out and replace it with “?”. The purpose is to predict the value of “?”. After we obtain the prediction value called* $p_{u_{1},i_{1}}$, *the error of prediction is hence computed by* $|r_{u_{1},i_{1}}-p_{u_{1},i_{1}}|$. *Then, we restore the value of* $r_{u_{1},i_{1}}$ *and leave the next rating out. This process terminates until all ratings are left out and predicted*.

### MAE and RSME

Given a rating system, the MAE [30] of the predictors is computed by

$$\begin{array}{c} \text{MAE}\left(r,p\right)=\frac{\sum_{u\in U,i\in I,r_{u,i}\neq 0}\left|r_{u,i}-p_{u,i}\right|}{\left|\{\left\langle u,i\right\rangle |u\in U,i\in I,r_{u,i}\neq 0\}\right|}, \end{array}$$ (2)

where *p*_*u*,*i*_ is the prediction rating of user *u* for item *i*, and the RSME [30] is computed by

$$\begin{array}{c} \text{RSME}\left(r,p\right)=\sqrt{\frac{\sum_{u\in U,i\in I,r_{u,i}\neq 0}{\left|r_{u,i}-p_{u,i}\right|}^{2}}{\left|\{\left\langle u,i\right\rangle |u\in U,i\in I,r_{u,i}\neq 0\}\right|}}. \end{array}$$ (3)

They are widely used to evaluate the performance of recommender systems. Naturally, the lower the value of MAE and RSME, the better the performance of the recommender system.

### Popular similarities

Various popular similarities are employed in recommender systems.

#### PIP

PIP, consisting of three factors (i.e., Proximity, Impact, and Popularity), is defined as [21]

$$\begin{array}{cc} \text{PIP}(i_{j},i_{q})= & \sum_{u\in C_{i_{j},i_{q}}}\text{Pro}\left(r_{u,j},r_{u,q)}\times \text{Imp}\left(r_{u,j},r_{u,q}\right)\right)\times \text{Pop}\left(r_{u,j},r_{u,q}\right), \end{array}$$ (4)

where the detail calculation can be found in [21].

#### NHSM

NHSM, consisting of two factors (i.e., JPSS and URP), is defined as [22]

$$\begin{array}{c} \text{NHSM}\left(i_{j},i_{q}\right)=\text{JPSS}\left(i_{j},i_{q}\right)\times \text{URP}\left(i_{j},i_{q}\right), \end{array}$$ (5)

where the detail calculation can be found in [22].

#### Cosine

Cosine which focuses on the angle between two vectors of items is defined as [18]

$$\begin{array}{c} \text{Cosine}\left(i_{j},i_{q}\right)=\frac{\vec{r_{j}}\cdot \vec{r_{q}}}{|\vec{r_{j}}\left|\times \right|\vec{r_{q}}|}, \end{array}$$ (6)

where $\vec{r_{j}}={\left(r_{u_{1},j},r_{u_{2},j},\cdots ,r_{u_{m},j}\right)}^{T}$ is the rating vector of item *i*_*j*_.

#### PCC

PCC which considers the linear correlation between two ratings vectors is defined as [6, 19]

$$\begin{array}{c} \text{PCC}\left(i_{j},i_{q}\right)=\frac{\sum_{u\in C_{i_{j},i_{q}}}\left(r_{u,j}-{\bar{r}}_{j}\right)\left(r_{u,q}-{\bar{r}}_{q}\right)}{\sqrt{\sum_{u\in C_{i_{j},i_{q}}}{\left(r_{u,j}-{\bar{r}}_{j}\right)}^{2}}\times \sqrt{\sum_{u\in C_{i_{j},i_{q}}}{\left(r_{u,q}-{\bar{r}}_{q}\right)}^{2}}}. \end{array}$$ (7)

#### CPCC

CPCC based on PCC, which considers the impact of positive and negative ratings, is defined as [13]

$$\begin{array}{c} \text{CPCC}\left(i_{j},i_{q}\right)=\frac{\sum_{u\in C_{i_{j},i_{q}}}\left(r_{u,j}-r_{med}\right)\left(r_{u,q}-r_{med}\right))}{\sqrt{\sum_{u\in C_{i_{j},i_{q}}}{\left(r_{u,j}-r_{med}\right)}^{2}}\times \sqrt{\sum_{u\in C_{i_{j},i_{q}}}{\left(r_{u,q}-r_{med}\right)}^{2}}}, \end{array}$$ (8)

where *r*_*med*_ is the median of *R*. If the *R* = {1, 2, 3, 4, 5}, we have *r*_*med*_ = 3.

#### Jaccard

Jaccard is defined as the size of the intersection divided by the size of the union of the rating users [20]

$$\begin{array}{c} \text{Jaccard}\left(i_{j},i_{q}\right)=\frac{|I_{j}\cap I_{q}|}{|I_{j}\cup I_{q}|}, \end{array}$$ (9)

where *I*_*j*_ = {*u* ∈ *U*|*r*_*u*,*j*_ > 0} and *I*_*q*_ = {*u* ∈ *U*|*r*_*u*,*q*_ > 0}.

#### BC

BC, which measures similarity by means of two probability distributions, is defined as [25, 26]

$$\begin{array}{c} \text{BC}\left(i_{j},i_{q}\right)=\sum_{x\in R}\sqrt{P_{j,x}\times P_{q,x}}, \end{array}$$ (10)

where *P*_*j*,*x*_ is the probability distribution of the rating *x* in item *j*.

#### ES

Euclidean distance (ED) which is the real distance between two points in Euclidean space is defined as [27, 28]

$$\begin{array}{c} \text{ED}\left(i_{j},i_{q}\right)=\sqrt{\sum_{u\in C_{i_{j},i_{q}}}{\left(r_{u,j}-r_{u,q}\right)}^{2}}. \end{array}$$ (11)

In Fig 1, |*AB*| is ED(*A*, *B*).

Therefore, ES can be computed by

$$\begin{array}{c} \text{ES}\left(i_{j},i_{q}\right)=1-\frac{\text{ED}(i_{j},i_{q})}{{\text{ED}}_{max}}, \end{array}$$ (12)

where *ED*_*max*_ is defined as

$$\begin{array}{c} {\text{ED}}_{max}=\left(R_{max}-R_{min}\right)\sqrt{|C_{i_{j},i_{q}}|}, \end{array}$$ (13)

where *R*_*max*_ is the maximum value (e.g., 5) of rating set *R*, and *R*_*min*_ is the minimum one (e.g., 1).

### kNN-based CF approach

The type of CF schema includes memory-based and model-based [31, 32] methods. The kNN [33, 34] algorithm is one of the most fundamental CF recommendation techniques. Here we adopt the kNN-based CF approach to predict the ratings. One key to kNN algorithms is the definition of the similarity measures. Popular measures have been presented. The prediction value of *r*_*u*,*j*_ is computed as follows.

$$\begin{array}{c} p\left(u,j\right)={\bar{r}}_{i_{j}}+\frac{\sum_{q\in h}Sim\left(i_{j},i_{q}\right)\left(r_{u,i_{q}}-{\bar{r}}_{i_{q}}\right)}{\sum_{q\in h}\left|Sim\left(i_{j},i_{q}\right)\right|}, \end{array}$$ (14)

where h is set of neighbors, and *Sim*(*i*_*j*_, *i*_*q*_) is similarity of items *i*_*j*_ and *i*_*q*_.

## Integrating Triangle and Jaccard similarities

In this section, we first propose the definition of Triangle similarity. Then we define the TMJ, and presented complexity analysis. Finally, we present a running example of TMJ.

### Triangle

The Triangle similarity is defined by

$$\begin{array}{c} \text{Triangle}\left(i_{j},i_{q}\right)=1-\frac{\sqrt{\sum_{u\in C_{i_{j},i_{q}}}{\left(r_{u,j}-r_{u,q}\right)}^{2}}}{\sqrt{\sum_{u\in C_{i_{j},i_{q}}}r_{u,j}^{2}}+\sqrt{\sum_{u\in C_{i_{j},i_{q}}}r_{u,q}^{2}}}, \end{array}$$ (15)

whose value range is [0, 1], where 0 indicates $C_{i_{j},i_{q}}=\emptyset$. The bigger value of Triangle, the more similar they are.

With the perspective of geometry, Eq (15) also can be defined as follows.

$$\begin{array}{cc} \text{Triangle}(i_{j},i_{q}) & =\text{Triangle}\left(\vec{OA},\vec{OB}\right)=1-\frac{\left|AB\right|}{\left|OA|+|OB\right|}, \end{array}$$ (16)

where $\vec{OA}$ is the rating vector of *i*_*j*_, $\vec{OB}$ is the rating vector of *i*_*q*_.

Triangle considers both the length of vectors and the angle between them, so it is more reasonable than the angle based Cosine measure. For example, given the two vectors *A* = (5, 5, 5) and *B* = (1, 1, 1), the Cosine similarity is 1, which is contrary to common sense. In contrast, the Triangle similarity between them is 0.33, more in line with expectations.

### TMJ

However, Triangle only considers the co-rating users. To provide more information about non co-rating users, we further combine Jaccard measure to improve Triangle, hence obtain a new hybrid measure as follows.

$$\begin{array}{c} \text{TMJ}\left(i_{j},i_{q}\right)=\text{Triangle}\left(i_{j},i_{q}\right)\times \text{Jaccard}\left(i_{j},i_{q}\right), \end{array}$$ (17)

which is the multiplication of Triangle and Jaccard similarity.

### Complexity analysis

Let the number of users and items be *m* and *n*, respectively. According to Eqs (9), (15) and (17), the time complexity of item similarity computation of Jaccard, Triangle, and TMJ is *O*(*m*).

kNN is employed to find the nearest *k* neighbors for each item. Therefore, for one item, the time complexity of finding all neighbors is *O*(*mn*).

In the leave-one-out cross validation scenario, all ratings should be predicted and validated. Since the maximal number of ratings is *mn*, the time complexity of testing the whole dataset is *O*(*m*^2^ *n*^2^).

### A running example

Given a rating system by Table 1. First, the co-rating users is obtained as $C_{i_{1},i_{3}}=I_{1}\cap I_{3}=\left\{u_{1},u_{3},u_{5}\right\}$. Second, the Triangle similarity between *i*_1_ and *i*_3_ is computed by

$$\begin{array}{ll} \text{Triangle}\left(i_{1},i_{3}\right) & =1-\frac{\sqrt{{\left(4-5\right)}^{2}+{\left(4-3\right)}^{2}+{\left(4-3\right)}^{2}}}{\sqrt{4^{2}+4^{2}+4^{2}}+\sqrt{5^{2}+3^{2}+3^{2}}}\\ & \approx 0.872. \end{array}$$

The Jaccard similarity between *i*_1_ and *i*_3_ is computed by

$$\begin{array}{cc} \text{Jaccard}(i_{1},i_{3}) & =\frac{I_{1}\cap I_{3}}{I_{1}\cup I_{3}}=\frac{3}{5}=0.6. \end{array}$$

Finally, the TMJ similarity between *i*_1_ and *i*_3_ is computed by

$$\begin{array}{cc} \text{TMJ}(i_{1},i_{3}) & =\text{Triangle}\left(i_{1},i_{3}\right)\times \text{Jaccard}\left(i_{1},i_{3}\right)=0.872\times 0.6=0.523. \end{array}$$

## Experiments

In this section, quality measures like the MAE, the RSME are applied to evaluate the above 10 similarity measures. Experiments are undertaken on four real world datasets such as MovieLens 100K, MovieLens 1M, FilmTrust and EachMovie.

### Datasets

In the experiments we used four real world datasets such as MovieLens 100K, MovieLens 1M, FilmTrust and Each Movie. The dataset schema is as follows.

- User (userID, age, gender, occupation)
- Movie (movieID, release-year, genre)
- Rating (userID, movieID)

We used the MovieLens 100K (943 users× 1,682 movies), MovieLens 1M (6,040 users × 3,952 movies), FilmTrust (1,508 users × 2,071 movies), and EachMovie (72,916 users × 1,628 movies). The detail of these datasets are shown in Table 2. However, 0 is a rating level in EachMovie dataset.

**Table 2:** Summaries of datasets.

Dataset	|*U*|	|*I*|	Ratings	Scale
MovieLens 100K	943	1,682	{1, 2, 3, 4, 5}	10^5^
MovieLens 1M	6,040	3,952	{1, 2, 3, 4, 5}	10^6^
FilmTrust	1,508	2,071	{0.5, 1, 1.5, …, 4}	10^5^
EachMovie	72,916	1,628	{0, 0.2, 0.4, 0.6, 0.8, 1}	10^6^

### Comparison of the MAE

Table 3 compares the MAE obtained by recommender systems using 10 similarity measures. Symbol “–” indicates that the algorithm cannot be completed within an acceptable period of time when the measure is used. The recommender system using the TMJ measure achieves the best/minimal MAE. In these four datasets, it is lower by 0.4%- 5.7%, 0.3%- 13.7%, 0.3%- 23.8%, and 0.1%- 5.5%, respectively, than the values obtained by other methods. The MAE of Triangle is also acceptable. It ranked fourth in the first dataset and third in the other three.

**Table 3:** The MAE comparison.

Measure/Dataset	MovieLens 100K	MovieLens 1M	FilmTrust	EachMovie
ES	0.764	0.808	0.852	0.234
BC	0.735	0.704	0.643	0.191
PCC	0.735	0.695	0.656	0.185
CPCC	0.731	0.694	0.657	0.186
Cosine	0.732	0.696	0.625	0.187
PIP	0.729	0.704	0.625	0.185
NHSM	0.718	–	0.617	–
Jaccard	0.711	0.674	0.617	0.180
Triangle	0.724	0.688	0.621	0.183
TMJ	**0.707**	**0.671**	**0.614**	**0.179**

**Fig 2:**
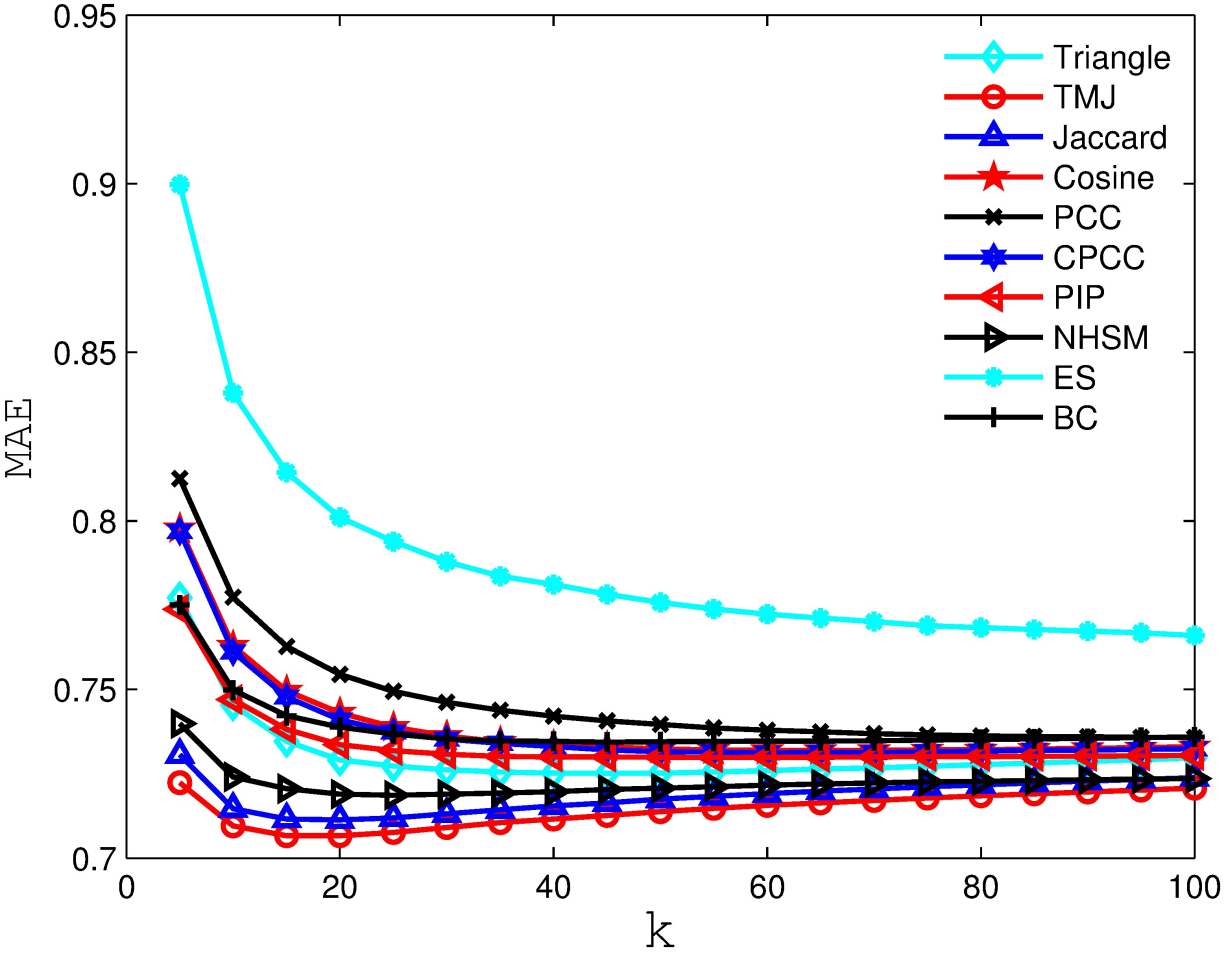
The MAE obtained by the recommender system using different similarity measures on MovieLens 100K.

**Fig 3:**
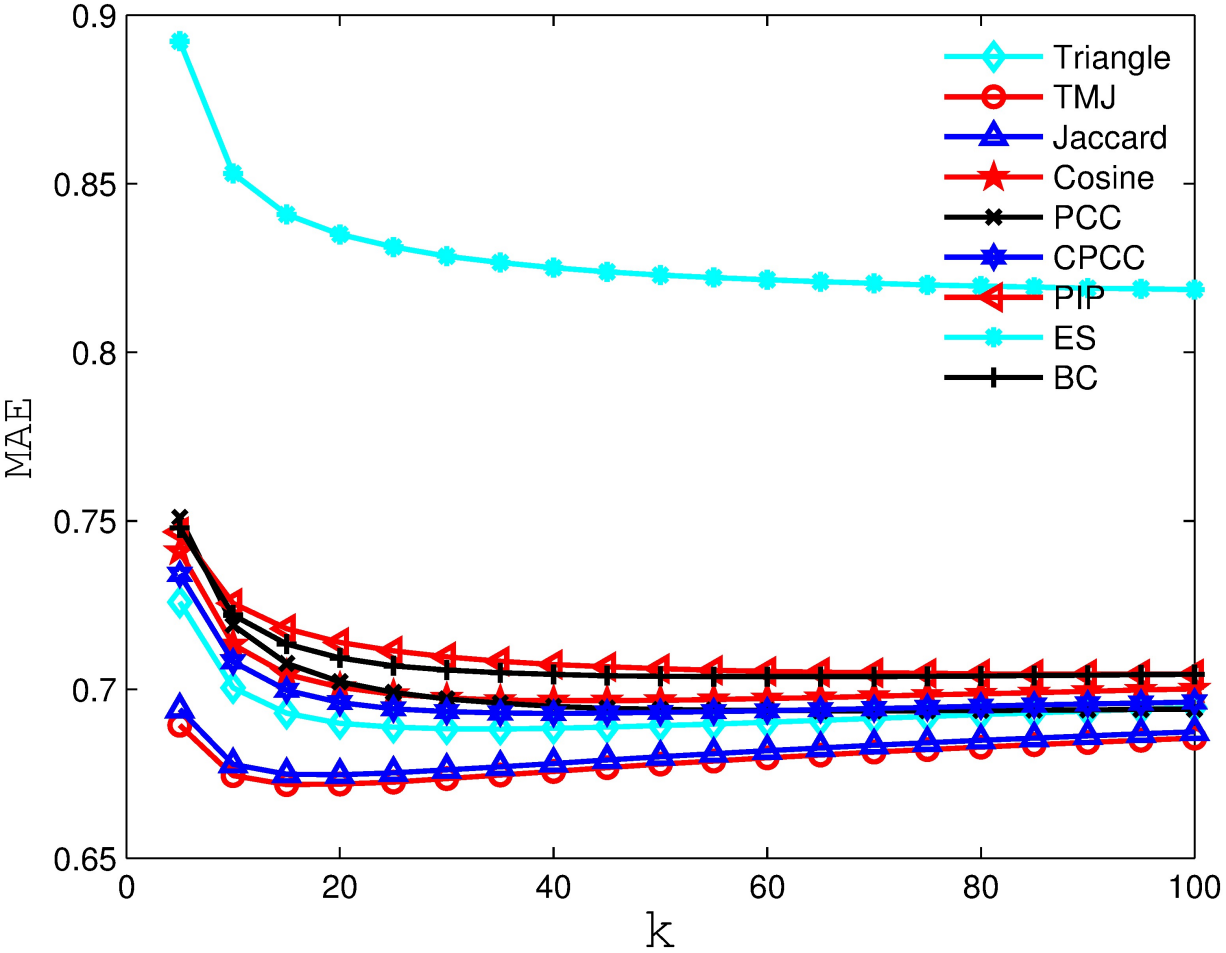
The MAE obtained by the recommender system using different similarity measures on MovieLens 1M.

**Fig 4:**
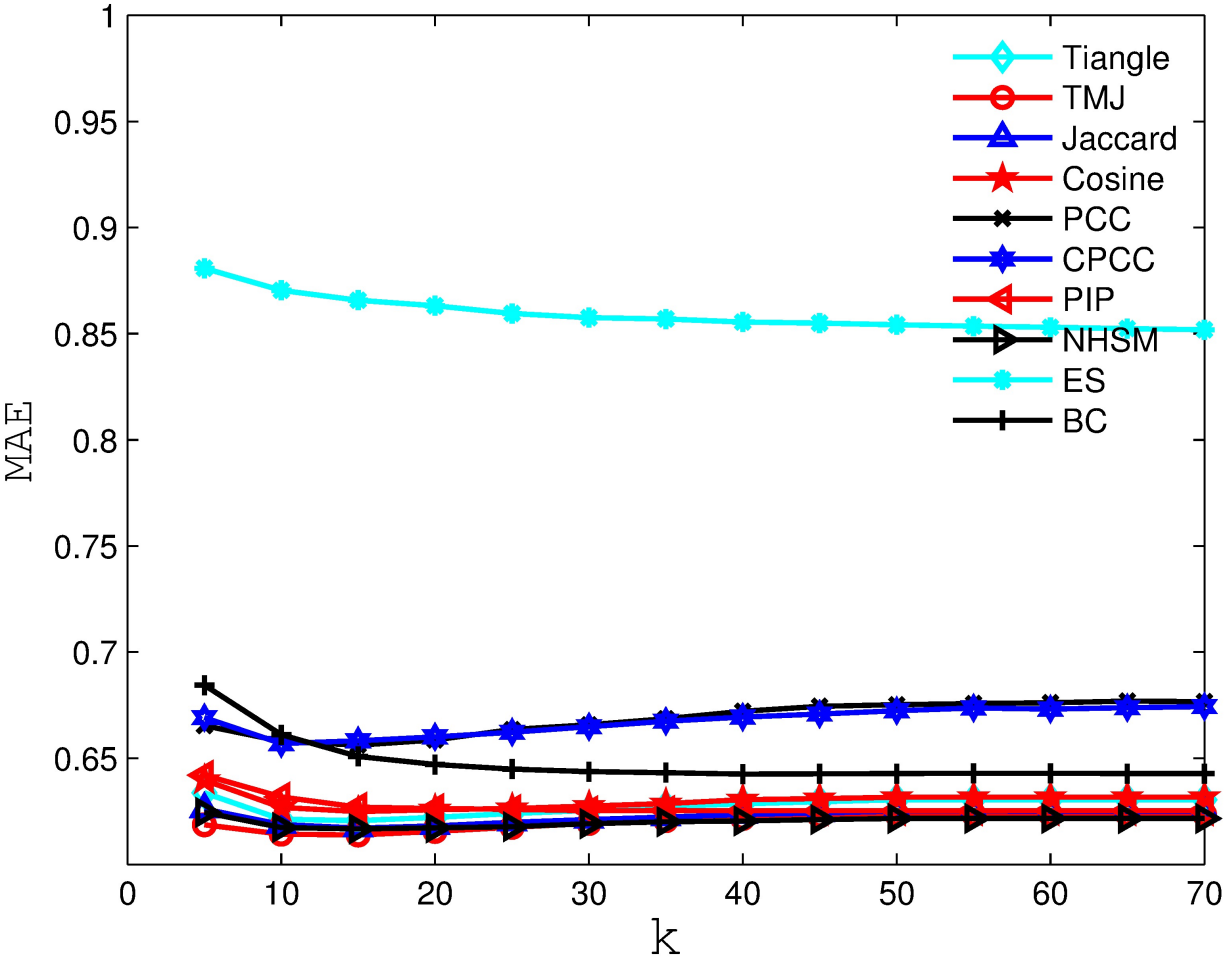
The MAE obtained by the recommender system using different similarity measures on FilmTrust.

**Fig 5:**
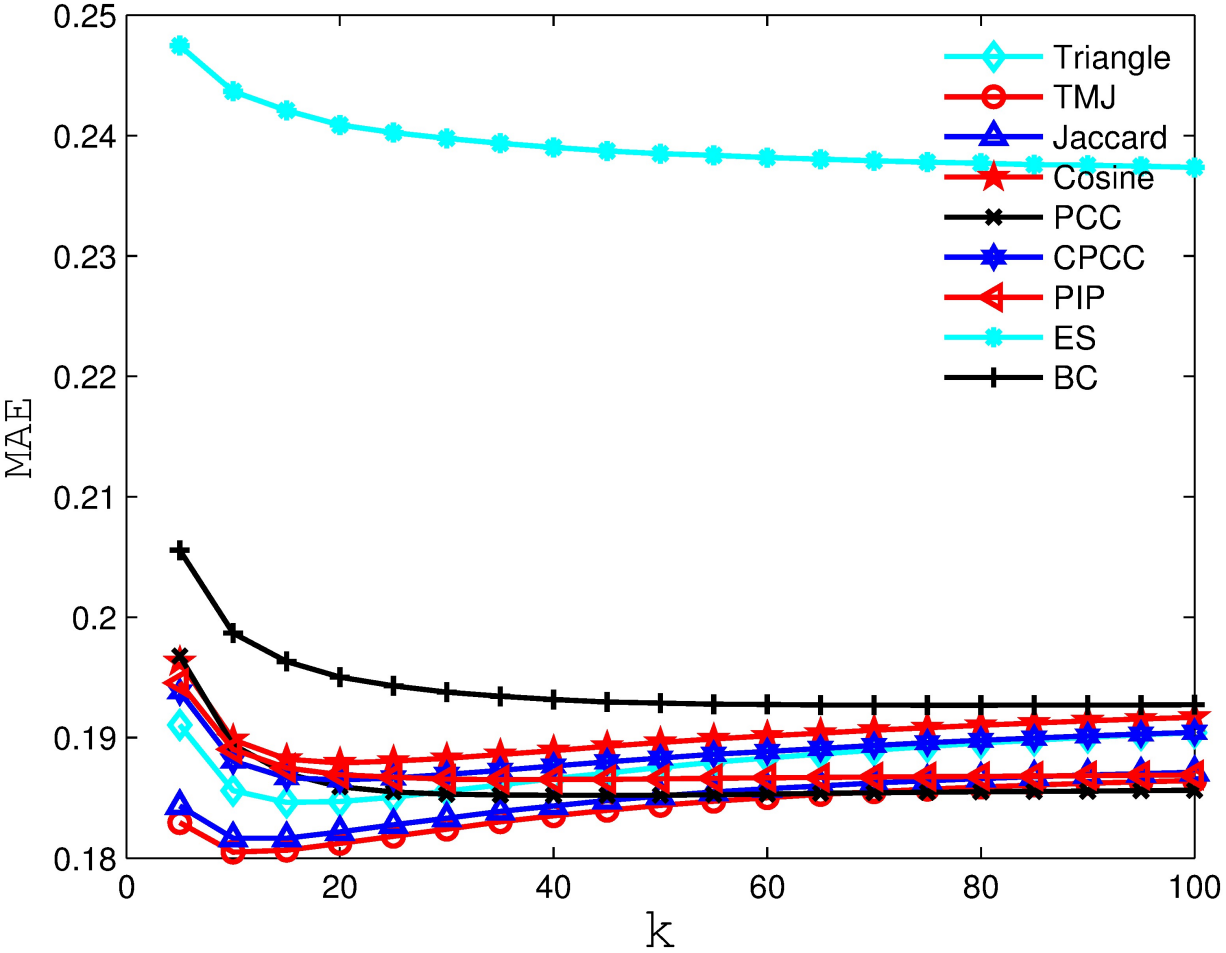
The MAE obtained by the recommender system using different similarity measures on EachMoive.

Figs 2, 3, 4 and 5 compare the MAE obtained by the recommender system using different similarity measures and setting different *k* values (i.e., number of the nearest neighbors). As we can see from the figure, the recommender system always obtains the best MAE when using TMJ, regardless of the *k* value. However, it obtains the best MAE, when *k* on the four datasets are 15, 15, 10, and 15, respectively.

### Comparison of the RMSE

Table 4 compares the RSME obtained by recommender systems using 10 similarity measures. Symbol “–” indicates that the algorithm cannot be completed within an acceptable period of time when the measure is used. The recommender system using the TMJ measure achieves the best/minimal RSME. In these four datasets, it is lower by 0.5%- 6.6%, 0.3%- 18%, 0.1%- 22.7%, and 0.1%- 6.1%, respectively, than the values obtained by other methods. The RSME of Triangle is also acceptable. It ranked fourth in the first dataset and third in the other three.

**Table 4:** RSME comparison.

Measure/Dataset	MovieLens 100K	MovieLens 1M	FilmTrust	EachMovie
ES	0.969	1.039	1.043	0.296
BC	0.934	0.895	0.838	0.248
PCC	0.937	0.889	0.903	0.240
CPCC	0.932	0.885	0.900	0.243
Cosine	0.931	0.886	0.829	0.243
PIP	0.926	0.893	0.823	0.240
NHSM	0.915	–	0.817	–
Jaccard	0.908	0.862	0.822	0.236
Triangle	0.923	0.877	0.821	0.240
TMJ	**0.903**	**0.859**	**0.816**	**0.235**

**Fig 6:**
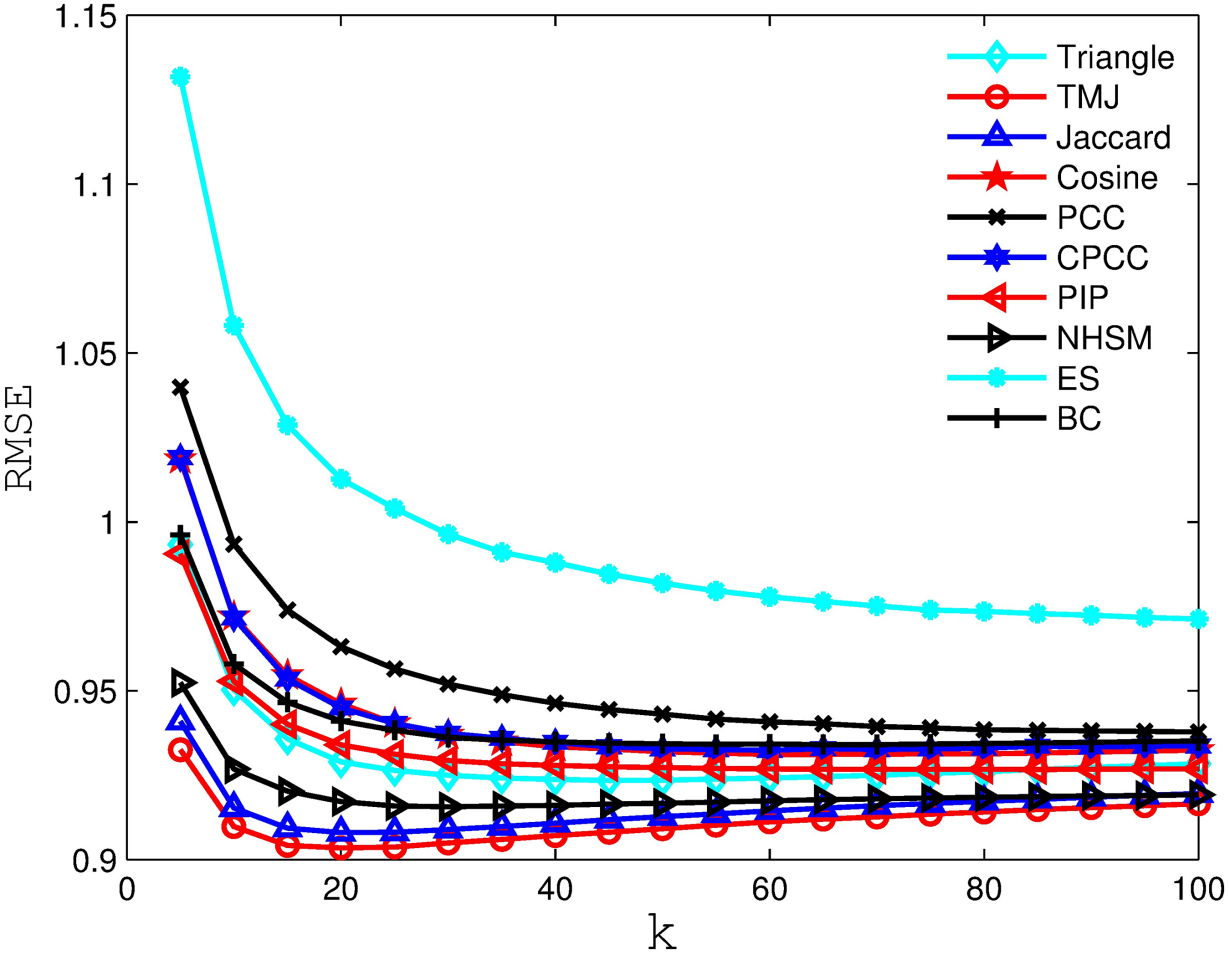
The RSME obtained by the recommender system using different similarity measures on MovieLens 100K.

**Fig 7:**
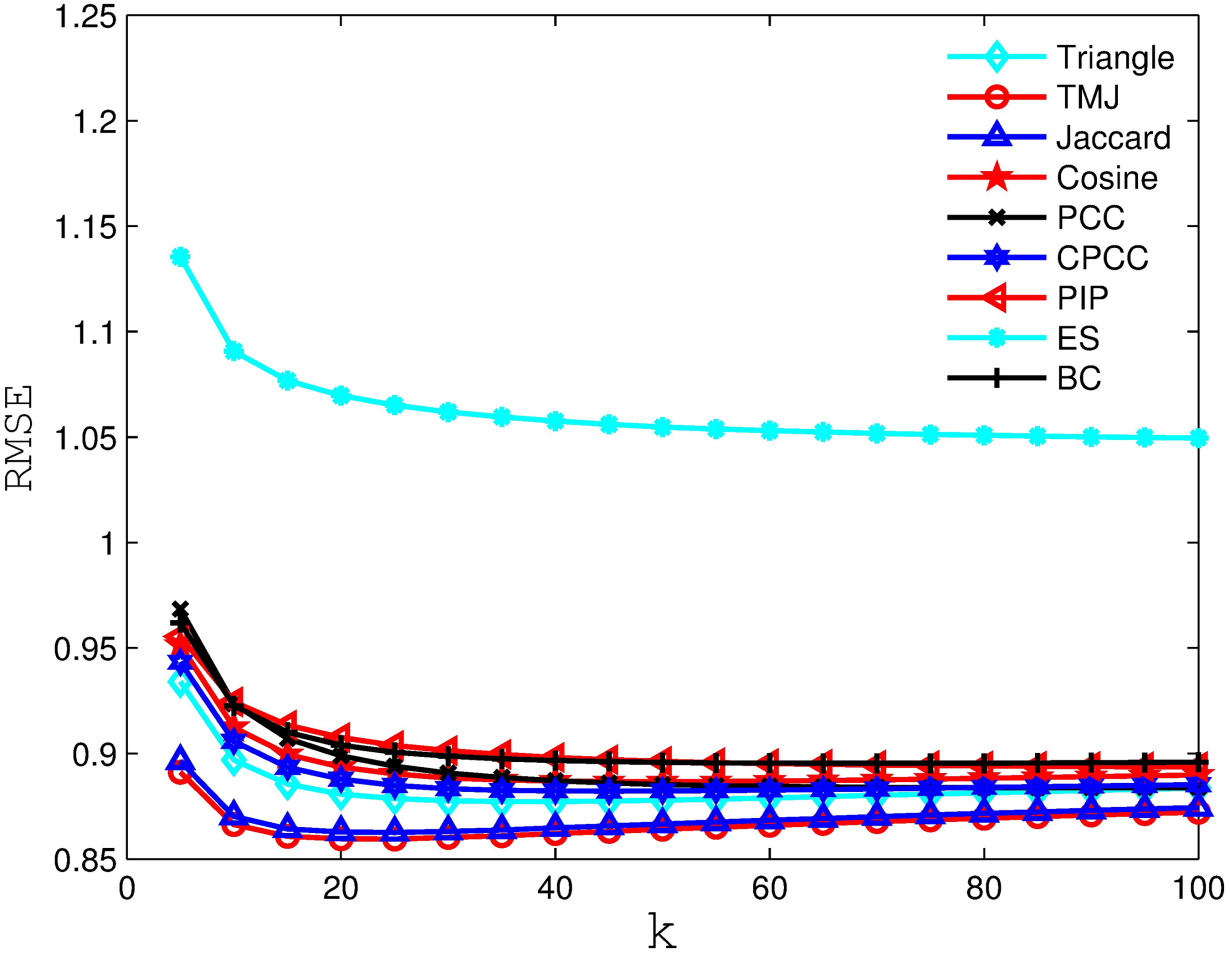
The RSME obtained by the recommender system using different similarity measures on MovieLens 1M.

**Fig 8:**
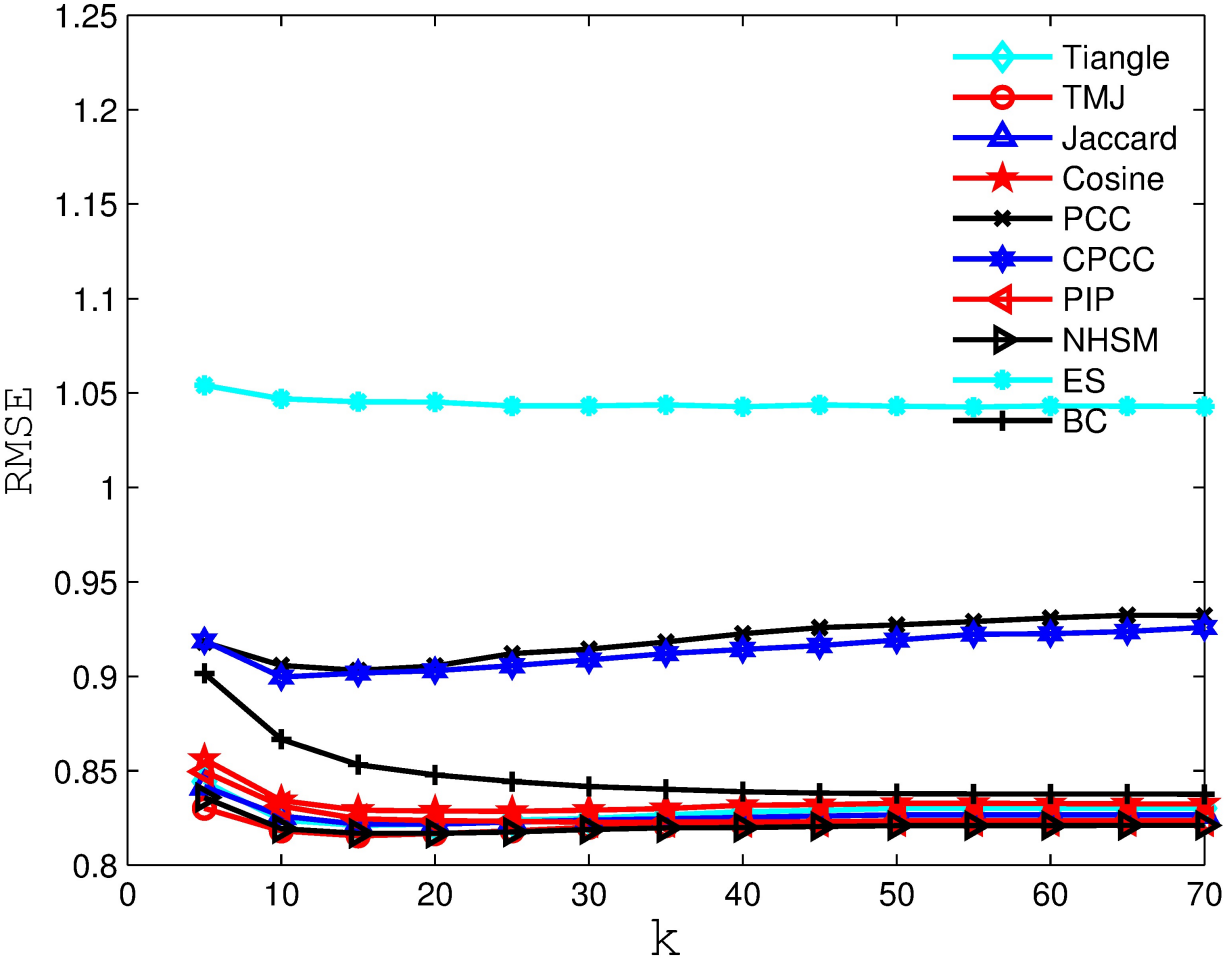
The RSME obtained by the recommender system using different similarity measures on FilmTrust.

**Fig 9:**
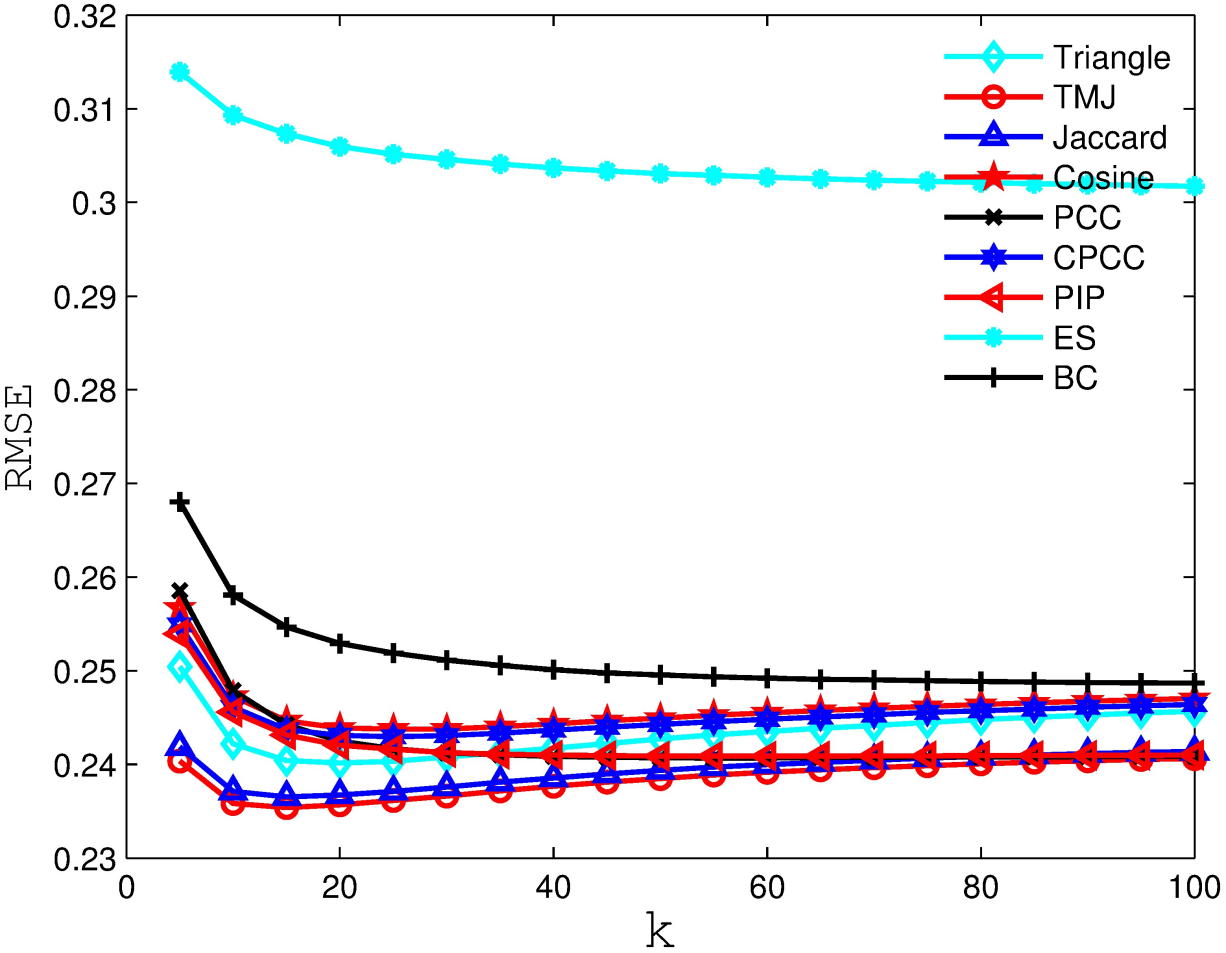
The RSME obtained by the recommender system using different similarity measures on EachMoive.

Figs 6, 7, 8 and 9 compare the RSME obtained by the recommender system using different similarity measures and setting different *k* values (i.e., number of nearest neighbors). As we can see from the figure, the recommender system always obtains the best RSME when using TMJ, regardless of the *k* value. However, it obtains the best RSME, when *k* on the four datasets are 15, 15, 10, and 15, respectively.

## Discussion

From the viewpoint of multiple kernel learning, the similarity measures such as Jaccard and Triangle meet the requirements of kernel function. TMJ is a product of Jaccard and Triangle. According to the property proved in [35] (pages 75–76), TMJ is also a kernel function.

There are various types of recommendation algorithms, such as kNN, NMF, LMF, etc. NMF algorithms address the recommendation task as the matrix completion problem with high sparsity. They intrinsically work in batch mode to predict all missing values. Since they do not need any similarity measure, we cannot incorporate our new measure into them. In fact, our new measure only serves as the basis of some similarity-based prediction models such as kNN. It can replace the existing measures anywhere, such as Manhattan, cosine, etc. In this sense it is general enough. However, support for batch mode is provided by the prediction model, rather than through the similarity measure. Hence we do not discuss this issue in more detail. To the best of our knowledge, kNN-based approaches usually predict rating one-by-one even for the split-in-two scenario.

## Conclusions

This paper defined the TMJ measure by integrating Triangle and Jaccard similarities. The new measure outperforms all the counterparts in terms of the MAE and the RMSE. In the future, we will apply the new measure to other tasks, such as the three-way recommendation [7, 36–42], clustering [2, 43], and image processing [5, 44, 45]. We will also develop other similarity measures in the light of multi-kernel learning [44, 46].
